# Ultrafast Nuclear Magnetic Resonance as a Tool to Detect Rapid Chemical Change in Solution

**DOI:** 10.1021/acsphyschemau.4c00042

**Published:** 2024-07-24

**Authors:** Ben. J. Tickner, Kawarpal Singh, Vladimir V. Zhivonitko, Ville-Veikko Telkki

**Affiliations:** † Department of Chemistry, University of York, Heslington, York YO10 5NY, United Kingdom; ‡ Department of Chemistry, University of Cambridge, Cambridge CB2 1EZ, United Kingdom; § NMR Research Unit, Faculty of Science, University of Oulu, Oulu 90570, Finland

**Keywords:** Ultrafast, magnetic resonance, spatial-encoding, Laplace, reactivity, hyperpolarization

## Abstract

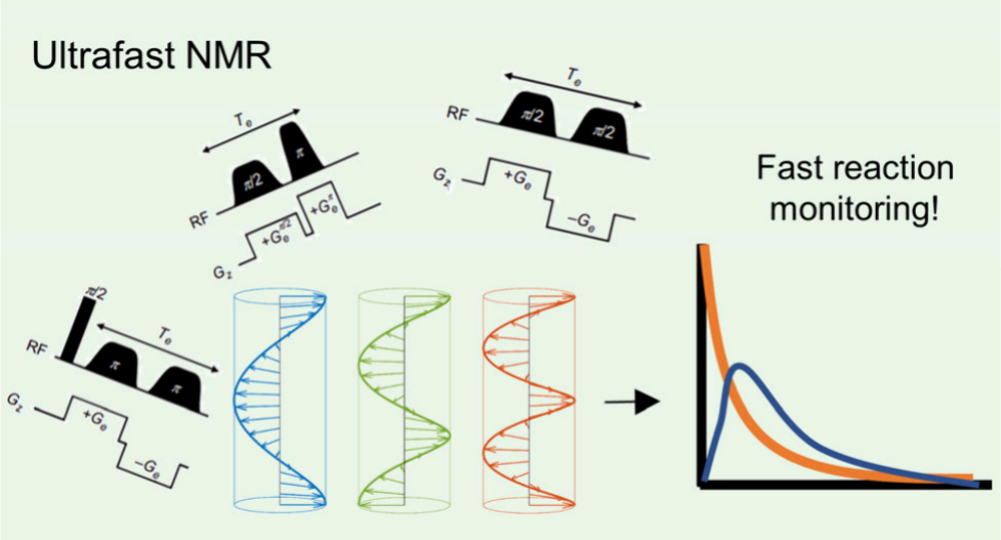

Ultrafast nuclear magnetic resonance (NMR) uses spatial encoding to record an entire two-dimensional data set in just a single scan. The approach can be applied to either Fourier-transform or Laplace-transform NMR. In both cases, acquisition times are significantly shorter than traditional 2D/Laplace NMR experiments, which allows them to be used to monitor rapid chemical transformations. This Perspective outlines the principles of ultrafast NMR and focuses on examples of its use to detect fast molecular conversions *in situ* with high temporal resolution. We discuss how this valuable tool can be applied in the future to study a much wider variety of novel reactivity.

## Introduction

Magnetic resonance (MR) is one of the most indispensable tools available to scientists, as it can provide detailed information about molecular structure, environment, and dynamics. MR methods are ideally suited to study chemical changes as they are noninvasive and do not destroy or ionize the samples being studied. Accordingly, MR, either in the form of nuclear magnetic resonance (NMR) spectroscopy or magnetic resonance imaging (MRI), receives widespread use across all scientific disciplines in both academia and industry.^1^ However, a significant weakness is associated with its inherently low sensitivity. MR can only detect a small fraction of the available nuclear spins (*i.e.*, 1 in 32,000 for ^1^H at 9.4 T) as nuclear spin states are close in energy and population differences across them are very small. This weakness is commonly addressed by using highly concentrated samples (>mM), signal averaging and paying the associated time cost to do this, and/or using spectrometers at higher field strength with higher equipment costs. Further challenges can arise from signal overlap in complex samples. This is most evident in ^1^H NMR, for which the entire chemical shift window typically spans just 10 ppm. Heteronuclear NMR experiments can provide a much larger chemical shift range, although X-nuclei have a lower NMR receptivity than ^1^H. Direct NMR detection of nuclei with high chemical importance but with low NMR receptivity takes significantly more time to record sufficient transients.

Higher order dimensional spectroscopy is often used to improve resolution and separate overlapping signals by adding an indirect dimension.^2^ These experiments typically rely on ^1^H-X through bond correlations or NOE through space correlations to record two-dimensional (2D) data sets. However, they can still require significant time to record, as they must be repeated *n* times to generate *n* points in the indirect dimension. Further repetitions of the 2D experiment may be needed for sufficient signal averaging to achieve sufficient signal-to-noise. Accordingly, 2D magnetic resonance is often ill-equipped to study the most rapid chemical transformations as a reaction may be completed by the time enough signal averaging has been performed to discern a signal. There is therefore a significant desire for methods that allow 2D data sets to be recorded rapidly. These fast NMR methods are highly compatible with studies of rapid chemical transformations due to their reduced acquisition times.

In this Perspective, we focus on ultrafast MR methods which use spatial encoding to record 2D data sets in a single scan. These approaches have only been developed in the last *ca*. 15–20 years and have been applied to study a limited number of rapid chemical transformations. We briefly explain the principles behind how these experiments work and give examples of how they have been applied to study novel reactivity. We also give our perspective on future directions of this research area including extension of the principles of spatially encoded ultrafast MR to Laplace NMR, and combination of ultrafast NMR with nuclear spin hyperpolarization to boost NMR signal intensities. Our aim is to stimulate a wider range of chemists to use ultrafast NMR experiments to monitor rapid reactivity.^3^ Here, we focus on the application of spatially encoded ultrafast NMR to study reactivity (although the technique can be used to study inert systems) and confine our examples to solution state spectroscopy, although we note that these methods can be applied to the solid state,^3^ and MRI.^4,5^ Accordingly, we do not go into significant detail regarding the necessary pulse sequences and direct any interested reader to other works that focus in detail on ultrafast pulse sequence design and implementation.^5−7^

## What Is Ultrafast NMR and How Does It Work?

**Figure 1:**
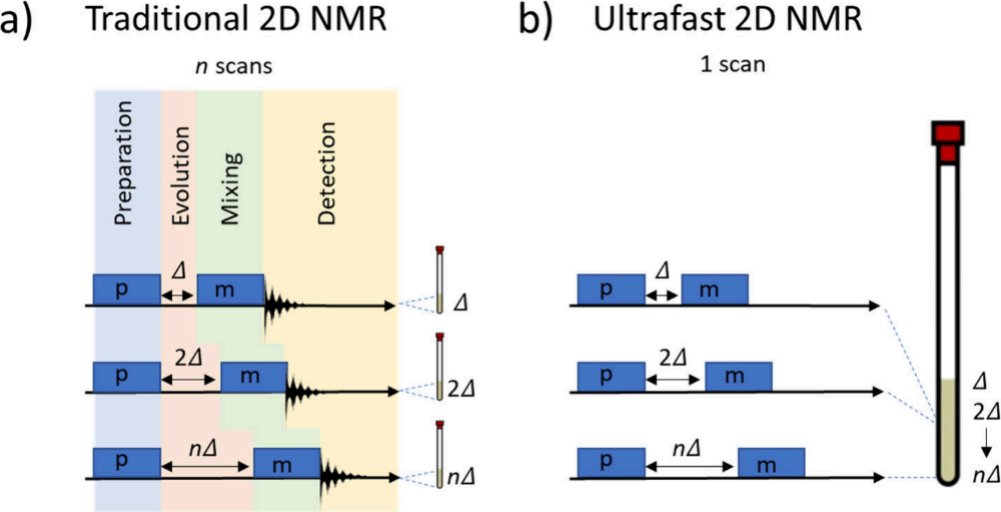
(a) Traditional 2D NMR involves preparation (*p*) as well as mixing (*m*) and detection periods (FID) separated by a variable evolution time (*t*_1_) with the experiment repeated *n* times for *n* different evolution times (*t*_1_ = Δ, 2Δ, ..., *n*Δ). (b) Spatially encoded ultrafast NMR allows different evolution times to be collected in just a single scan.

Simple 1D NMR involves radiofrequency excitation, followed immediately by spectral acquisition. 2D NMR offers enhanced resolution by discerning distinct resonances while furnishing additional insights into the molecular structure and dynamics. Multidimensional NMR differs from 1D NMR as the preparation stage is now separated from the signal detection by a variable time evolution delay and a mixing period (Figure 1a). To this end, 2D NMR sequences involve radiofrequency excitation pulses to prepare coherences, followed by some evolution delay to allow the coherence to evolve.^1,2^ This delay is followed by a second block called the mixing period, during which, e.g., magnetization is transferred from one nucleus to another, after which the signal is detected. 2D data sets are generated by varying the length of the evolution time between the preparation and mixing blocks. Therefore, 2D experiments must be repeated *n* times with *n* indicating different values of the evolution time. The design of the pulses in the preparation stage will depend on the specific type of 2D NMR experiment. Traditional 2D NMR experiments rely on a sequential approach involving a series of subexperiments with incremented evolution periods. Typically, the evolution delay is incremented from tens to hundreds of times with an appropriate relaxation delay (typically on the order of seconds) between the increments, and therefore conventional 2D NMR takes on the order of minutes for each scan. This time penalty is particularly limiting for samples undergoing chemical or biochemical changes, where their composition or structure alters.

Several different strategies can be employed to reduce 2D acquisition times.^8^ One approach is to rely on non-uniform sampling methods to reduce the time required to collect points in the indirect dimension.^9−12^ These methods sample just a portion of indirect dimension points with nonconsecutive time increments, rather than the traditional recording of equally spaced indirect evolution time increments. Sampling strategies are often complex as they must balance heavy sampling of initial data points (where the signal is strongest) for increased sensitivity with the need to randomly sample indirect points for minimization of spectral artifacts. Other strategies include Hadamard NMR spectroscopy^13−15^ which involves *n* simultaneous selective radiofrequency pulses for *n* distinct chemical shifts. The time required to record a 2D data set is reduced as data for sparse or empty regions of the spectra are not recorded, although it relies on the prior knowledge of the n distinct chemical shifts. Other NMR pulse sequences have also been developed to speed up spectral acquisition using a combination of selective manipulation of ^1^H spin subsets to shorten *T*_1_ relaxation times and optimized flip angles to increase repetition rates and consequently speed up data acquisition.^16−20^ Experiments of this type (e.g., SOFAST-HMQC) have been used to record 2D NMR spectra for proteins in just a second, and has even been employed to monitor deuteration of protein amide sites.^11^

In this Perspective, we focus on NMR methods that exploit spatial encoding to facilitate single scan data collection, and these are often collectively called “ultrafast NMR”. Application of magnetic field gradients can allow diffusion,^21^ relaxation,^22,23^ or NOE^24^ measurements to be made in a single scan. Similarly, they can be used in conjunction with 2D experiments such as HSQC,^25^ HMBC,^26^ COSY,^27^ TOCSY,^28^ and SQ/DQ^29−32^ to record the entire data set in just a single scan as they no longer require *n* repetitions to collect points in the indirect dimension.^33^ This innovative approach involves simultaneous acquisition of multiple evolution time increments of an NMR experiment rather than sequential acquisition (Figure 1b). Accordingly, as the entire data set is recorded in just a single scan taking on the order of a few seconds, the acquisition times are significantly compressed, even if the experiments are repeated several times for signal averaging. Single-scan acquisition of 2D data sets is achieved using similar principles to MRI, in which application of an external magnetic field gradient along the axis of the magnet bore (*z*) and parallel to the direction of the magnetic field (B_0_) encodes spatial position.^34^ By using a spatial parallelization scheme, different slices of the NMR tube can be manipulated independently, with each slice corresponding to experiments with different mixing times (Figure 1b). Therefore, there is no longer the requirement for *n* repetitions in ultrafast experiments.

**Figure 2:**
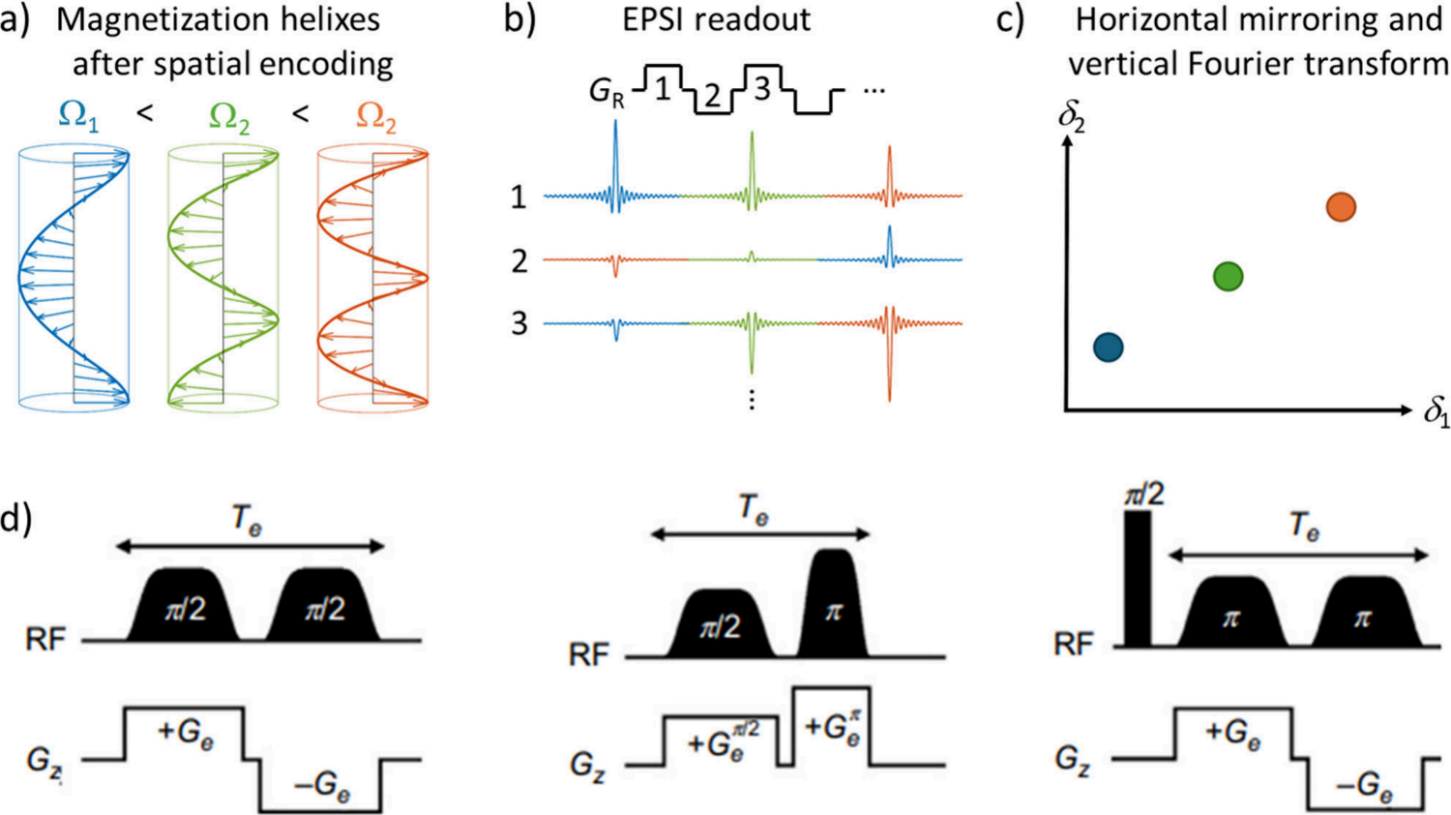
(a) Spatial encoding is achieved using chirp pulses and magnetic field gradients. After spatial encoding, magnetization vectors at different layers form helixes, as the evolution time in ultrafast NMR experiments is linearly dependent on position. The tightness (pitch length) of the helix depends on the chemical shift of the spins. Helixes for three different representative chemical shifts (Ω_*i*,_*i* = 1–3) are shown. (b) Magnetization helixes are unwound by a readout gradient *G*_R_. First, the loosest helix corresponding to the smallest chemical shift is unwound, producing an echo. Thereafter, echoes of other spins are observed in the order of their chemical shifts. Hence, the time domain signal is identical with a conventional spectrum without any Fourier transform. The helixes are unwound and rewound repeatedly by using gradients with opposite polarity in order to observe time evolution in the direct (EPSI) dimension. (c) Mirroring the time domain signal corresponding to negative readout gradients and Fourier transform along the EPSI (vertical) dimension results in a 2D spectrum measured in a single scan. (d) Examples of pulse sequence blocks used to achieve spatial encoding. In an ultrafast NMR experiment, these blocks are combined with preparation and mixing periods similar to conventional 2D NMR experiments as well as EPSI-type detection. Panel (d) is reproduced and adapted with permission from ref (7). Copyright 2018, Elsevier.

Generating a 2D NMR spectrum using ultrafast NMR entails two crucial steps: implementing spatial encoding and subsequently decoding the information during the acquisition period. The acquired data are then utilized to reconstruct the 2D NMR spectrum. The magnetization vectors of a group of spins are initially in thermal equilibrium along the *z*-axis. These can be tipped into the xy plane after application of a hard 90° excitation pulse. Typical hard excitation pulses used in NMR have a fixed phase and fixed frequency, and their bandwidths (the range of frequencies excited by the pulse) are defined by their duration. In contrast, ultrafast NMR experiments use chirp pulses and magnetic field gradients to achieve spatial encoding. Chirp radiofrequency pulses^35^ have a frequency that varies as a function of time and, to a good approximation, they only affect spins whose precessional frequencies equal the frequency of the chirp pulse. In this way, by linearly sweeping the frequencies, it is possible to sequentially excite all frequencies. Therefore, one-to-one matching between the chirp pulse frequency and spatial position is achieved when a chirp pulse is applied with a gradient pulse. In ultrafast NMR experiments, chirp and gradient pulses are designed so that the evolution time becomes linearly dependent on position *z* (Figure 1b), which means that the phases of spins at different layers have different times to evolve, and the phase also becomes linearly dependent on position, taking a minimum value at the top and a maximum at the bottom of the encoding layer. Consequently, after spatial encoding, the magnetization vectors form a helix, as illustrated in Figure 2a. As the phase is also dependent on the chemical shifts, spins with higher chemical shifts form a tighter helix, meaning that the pitch length of the helix becomes dependent on the chemical shift (Figure 2a). In principle, the chirp and gradient pulses induce additional phases for the magnetization vectors at different layers. However, these undesired phases are canceled out by applying pairs of chirp pulses and gradients. There are several different spatial encoding schemes used to achieve the desired chemical shift (or some other spectral parameter) dependent magnetization helixes, all of which involve application of two chirp pulses with gradients (Figure 2d).^36,37^

Regardless of the spatial encoding scheme, it is followed by a mixing period, which is similar to that in conventional 2D NMR experiments. After mixing, the signal is detected with the presence of a readout gradient. The positive readout gradient unwinds the loosest helix first, and consequently the echo corresponding to the spins with the smallest chemical shift is observed (blue signal in trace 1 in Figure 2b). The echoes of the other spins are observed in the order of their chemical shifts. Therefore, the time domain signal is analogous to the conventional NMR spectrum without any Fourier transform. The magnetization helixes are unwound and rewound several times using gradients with opposite polarities similar to echo planar spectroscopy imaging (EPSI).^38,39^ The same spectrum is observed each time, although it is mirrored when negative gradient readings are used. However, the amplitudes of the signals are modulated due to time evolution. Hence, mirroring the time domain signals corresponding to the negative read gradient (horizontal traces 2, 4, . . . in Figure 2b) and applying a Fourier transform along the direct (vertical) direction results in a 2D spectrum measured in a single scan (Figure 2c).

While spatially encoded 2D experiments can be recorded quickly, they often do not have the resolution of conventional 2D NMR experiments. Spatially encoded ultrafast NMR records signals in the presence of strong magnetic field gradients, which are applied parallel to the applied magnetic field (B_0_, *z* axis). A consequence of this is that large digital filter bandwidths are required which has an effect on signal-to-noise and results in a sensitivity penalty associated with spatial encoding.^40,41^ This sensitivity loss can be influenced by other factors such as diffusion or *J*-coupling during spatial encoding and can be limited by reducing the time spins spend in the transverse plane (the echo time), using weaker excitation gradients, viscous solvents to reduce molecular diffusion and multiple-echo encoding schemes.^42,43^ While these experiments provide an obvious time advantage, their limitations compared to traditional multidimensional NMR is that they can be challenging to set up and optimize. Ultrafast experiments can suffer from *J*-modulated artifacts or peak folding associated with the narrow bandwidth of chirp pulses used for selective spatial excitation.^44^ Encoding and decoding of the entire chemical shift window can also be a challenge as high gradient strengths are required to do this, although approaches such as interleaved acquisition^45,46^ or recording spectra for particular aliphatic, olefinic or aromatic windows can be employed.^25^ It is also worth noting that ultrafast experiments are often analyzed using custom-made codes rather than standard spectrometer software. For all of these reasons, ultrafast experiments are not currently used as routine experiments, although their use is increasing.

Most conventional and ultrafast 2D NMR experiments are typically utilized for qualitative detection. This is because, unlike the peak integrals from most 1D experiments, 2D peak volumes are influenced by nonconcentration factors including *J*-coupling and relaxation times. Despite this, quantitative information from 2D measurements can be extracted in some cases, as 2D peak volumes usually contain a linear dependence on concentration. Therefore, if appropriate calibration is performed, which usually requires separate calibration for each 2D peak of interest, the different contributions of *J*-coupling and relaxation on each 2D peak can be effectively accounted for.^47^ Accordingly, conventional 2D NMR and spatially encoded ultrafast NMR have shown great promise for quantitative detection, which is highly useful for reaction monitoring applications.

## Using Ultrafast NMR to Study Rapid Chemical Reactions in Real Time

**Figure 3:**
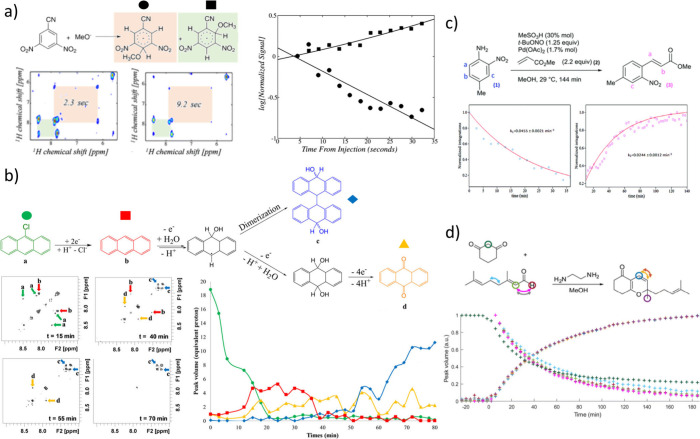
(a) Reaction of 1-cyano-3,5-dinitrobenzene with methoxide. Two examples of ultrafast TOCSY spectra are also shown at the indicated reaction times, which allow the two products to be readily distinguished. The volume of 2D cross peaks allows concentration to be determined and monitored over time (right) and reveals that the kinetic product formed from substitution at the 4 position is favored at short reaction times, before the thermodynamic product formed from substitution at the 2 position dominates at longer reaction times. These isomeric products could not be distinguished using ^1^H NMR spectroscopy. Reproduced and adapted with permission from ref (48). Copyright 2006, American Chemical Society. (b) Reduction of of 9-chloroanthracene *via* an unstable anthracene intermediate to form anthraquinone and the major product 10,10′-dihydroxy-9,9′,10,10′-tetrahydro-9,9′-bianthryl. The reaction was monitored using ultrafast COSY inside an electrochemical cell situated inside an NMR tube. Example spectra are shown on the left, with the corresponding reaction time course on the right. Reproduced and adapted with permission from ref (27). Copyright 2015, American Chemical Society. (c) Palladium-catalyzed Heck-Matsuda cross coupling was monitored using ultrafast COSY spectra on a 1T benchtop NMR spectrometer, allowing a reaction time course to be extracted which could be fitted to a kinetic model to determine rate constants. Reproduced and adapted with permission from ref (52). Copyright 2015, Royal Scoiety of Chemistry. (d) Reaction between citral and 1,3-cyclohexanedione was monitored using ultrafast COSY and the reaction followed using a continuous flow setup involving a flow cell inserted into a classical NMR probe at 11.7 T. Reproduced and adapted with permission from ref (53). Copyright 2023, Royal Society of Chemistry.

One of the earliest examples of chemical reactions studied using ultrafast NMR was the reaction of 1-cyano-3,5-dinitrobenzene with methoxide.^48^ In this reaction, methoxide addition can occur to either the 2 or 4 position of the phenyl ring (Figure 3a). The three coupled protons in each of these two isomeric products could not be readily distinguished using ^1^H NMR spectroscopy but could be using TOCSY experiments. However, due to the speed of this reaction, the time required to record one traditional 2D TOCSY data set (>minutes) is significantly longer than the time frame of the reaction (seconds). Therefore, a series of consecutive spatially encoded ultrafast TOCSY measurements were employed (Figure 3a). This allowed 12 separate TOCSY data sets to be collected during a 30 s time window. As the integral intensity of the TOCSY cross peaks relates to species concentration, collection of a series of these ultrafast 2D measurements allowed concentration changes over highly compressed reaction times to be extracted. Therefore, the amounts of the two isomeric products formed in the reaction could be monitored over a period of just 30 s. Consequently, ultrafast TOCSY could reveal that the kinetic product formed from methoxide addition to the 4 position is present at very short reaction times, before it is replaced by the thermodynamically favored product formed from addition to the 2 position (Figure 3a). These reaction dynamics would be challenging to extract from traditional 1D NMR experiments which could not differentiate the isomeric products, or conventional 2D NMR which would only be able to detect the thermodynamic product as long acquisition time prevents data sets being recorded at very short reaction times.

Since then, the number of examples of rapid reactions monitored using spatially encoded ultrafast NMR have increased. It has been used to monitor organic reactions,^25,49,50^ electrochemical reactions of relevance to battery studies,^27^ and has even been used to study reactions on benchtop NMR spectrometers^51,52^ and processes in continuous flow.^53^ Redox reactions, which involve the transfer of electrons between molecules, are highly useful in electrochemistry and have applications in battery technology that rely on electron transfer between donors and acceptors. These reactions often occur *in situ* and to study them using a noninvasive technique such as magnetic resonance is highly attractive. As redox reactions can be extremely rapid, they can be challenging to probe using traditional NMR. In one example, ultrafast 2D COSY experiments were used to examine the electrochemical reduction of 9-chloroanthracene inside an electrochemical cell situated inside an NMR tube.^27^ Reduction to an unstable anthracene intermediate and formation of anthraquinone, and the major reaction product 10,10′-dihydroxy-9,9′,10,10′-tetrahydro-9,9′-bianthryl could be monitored over an 80 min window, with a data set recorded every 3 min (Figure 3b). These ultrafast experiments proved superior to conventional COSY which could only be recorded every 10 min and did not detect observable correlation peaks as the species are chemically changing during the longer spectral acquisition.

Other types of ultrafast 2D experiments have also been used, for example, ultrafast TOCSY^28^ and HMBC^26^ experiments have been used to monitor the reaction between ketones and nitriles to form pyrimidines. In one case, 525 separate TOCSY experiments could be acquired at 10 s intervals.^28^ In the case of ultrafast HMBC spectroscopy, chemical changes at the carbonyl site were readily discerned.^26^ Both sets of experiments allowed the full kinetic profile of this rapid reaction, which is completed in around 15 to 20 min, to be discerned. Traditional 2D NMR would take around this time length to acquire just a single data point, thereby losing most of the valuable kinetic information at short reaction times that ultrafast NMR is able to capture.

In recent years, there has been a push toward low field NMR.^54^ This can be particularly useful for process monitoring as low field magnets are typically cheaper and smaller and can be portable. Therefore, they are more compatible for monitoring routine industrial chemical transformations and quality control. However, lower magnetic field strengths compress the frequency window and suffer from lower sensitivity. Nevertheless, ultrafast experiments can prove particularly valuable at low magnetic field as they may help to resolve peak overlap.^51^ In one example, 4-methyl-2-nitroaniline reacted with an alkene in a palladium-catalyzed Heck-Matsuda cross coupling to form a styrene (Figure 3c).^52^ A train of 55 ultrafast COSY spectra, with each taking 2.6 min to record, was used to monitor formation of the product at 1 T. The high temporal resolution allowed extraction of kinetic rate constants. In this case, the reaction could not be tracked using traditional 1D NMR spectra due to overlap of both reactant, product, and solvent peaks.

Other experimental setups have allowed reactions to be monitored in continuous flow mode, which is highly beneficial for the study of large-scale industrial reactions. An NMR flow cell inserted into a classical NMR probe at 11.7 T was used to monitor the condensation of 1,3-cyclohexanedione with citral using a series of ultrafast COSY spectra (Figure 3d).^53^

## Using Ultrafast NMR to Detect Short Lived Reaction Intermediates

**Figure 4:**
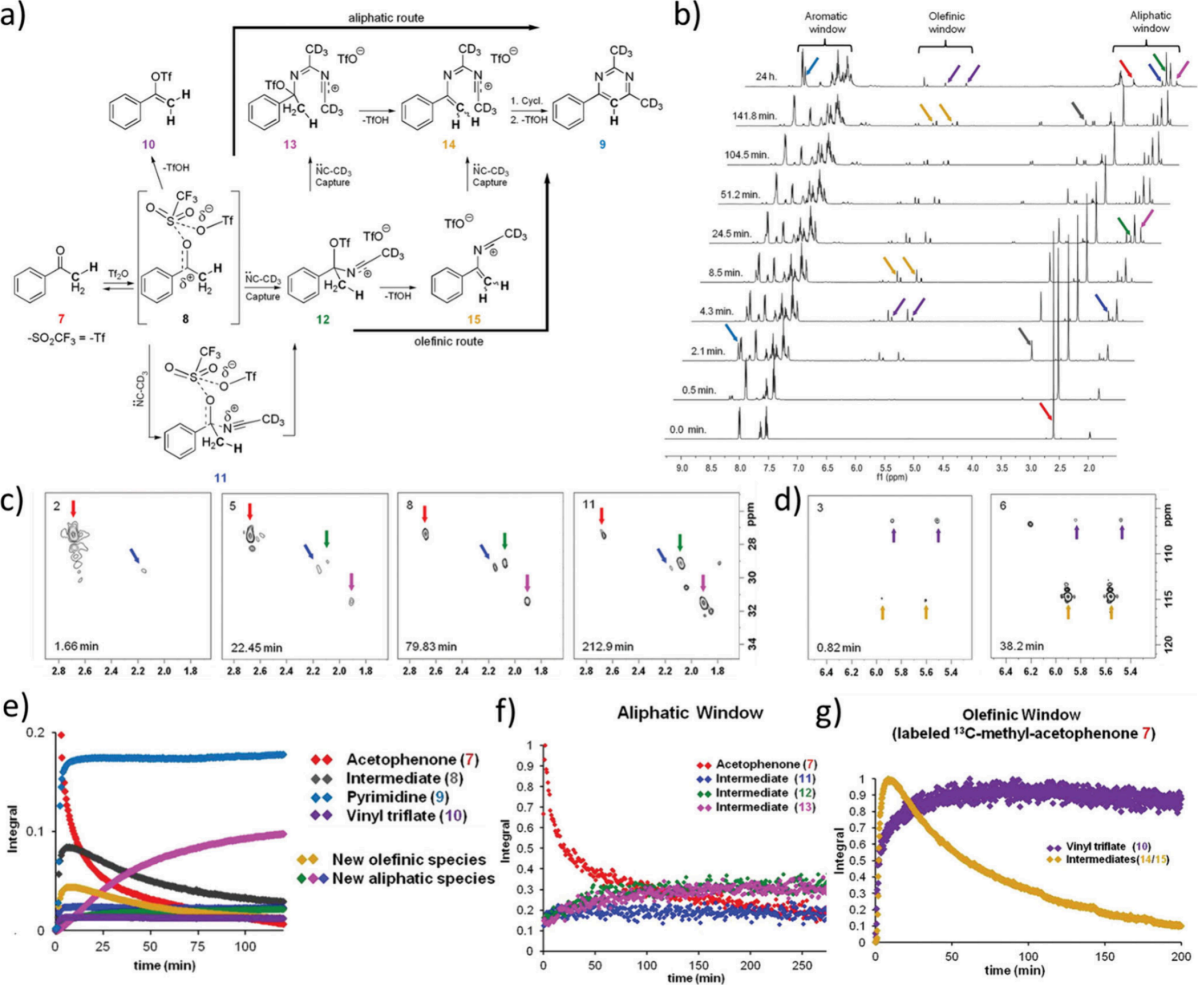
(a) Reaction of acetophenone with acetonitrile to form an alkyl pyrimidine was monitored using ultrafast NMR to reveal intermediates that are difficult to discern and characterize using traditional NMR methods. Accordingly, differing mechanistic pathways could be elucidated from the information provided from ultrafast NMR. The reaction was monitored using (b) a series of standard ^1^H NMR spectra and/or ultrafast HSQC detecting the (c) aliphatic region and (d) olefinic region. The associated kinetic time courses from these data are shown in (e)–(g), respectively. Reproduced and adapted with permission from ref (49). Copyright 2012, American Chemical Society.

While ultrafast NMR can extract kinetic time courses for rapid reactions, an added benefit associated with faster spectral acquisition is that many short-lived intermediates involved in chemical conversions can now be detected. In one such example, hydrolysis of an acetal to an aldehyde proceeds *via* a hemi acetal intermediate which is difficult to detect using standard NMR spectroscopy. However, when spatially encoded ultrafast HSQC spectra were recorded, correlations for the hemiacetal intermediate could in fact be observed and the 2D peak amplitude monitored during the course of the reaction.^25^ When similar ultrafast HSQC experiments were applied to study the formation of pyrimidines *via* reaction of ketones and nitriles, a series of novel short-lived intermediates could be detected, which gives valuable information about the mechanism of the reaction.^49^ For these experiments, ^13^C labeling of the carbonyl position was employed, which is costly and may not provide a general approach to study reactivity. Therefore, these reactions were also studied using ultrafast HSQC experiments on nonlabeled starting materials.^49^ The benefit of this approach is that spectra could be recorded over a wider spectral width, which is advantageous for detecting unknown side products or intermediates, whose chemical shifts may not be known beforehand. To record ultrafast spectra covering wide spectral widths would usually necessitate very high gradient strengths. However, this was alleviated by selective excitation of particular spectral windows (*i.e*., aromatic or aliphatic) and using interleaved acquisition.^49^ When these methods were applied to study the reaction between acetophenone and acetonitrile, they revealed the formation of pyrimidine and vinyl triflate products in addition to a range of intermediates (Figure 4). Excitingly, detection of these novel intermediates allowed two distinct mechanistic routes to be determined. This example shows the remarkable power of ultrafast 2D NMR as the *ca*. 100 min reaction could be monitored using up to 2500 separate 2D NMR experiments. As this feat cannot be achieved using traditional 2D NMR, signals from many of these intermediates are not discerned, and valuable mechanistic information is lost.

## Applying the Ultrafast Approach to Laplace NMR

**Figure 5:**
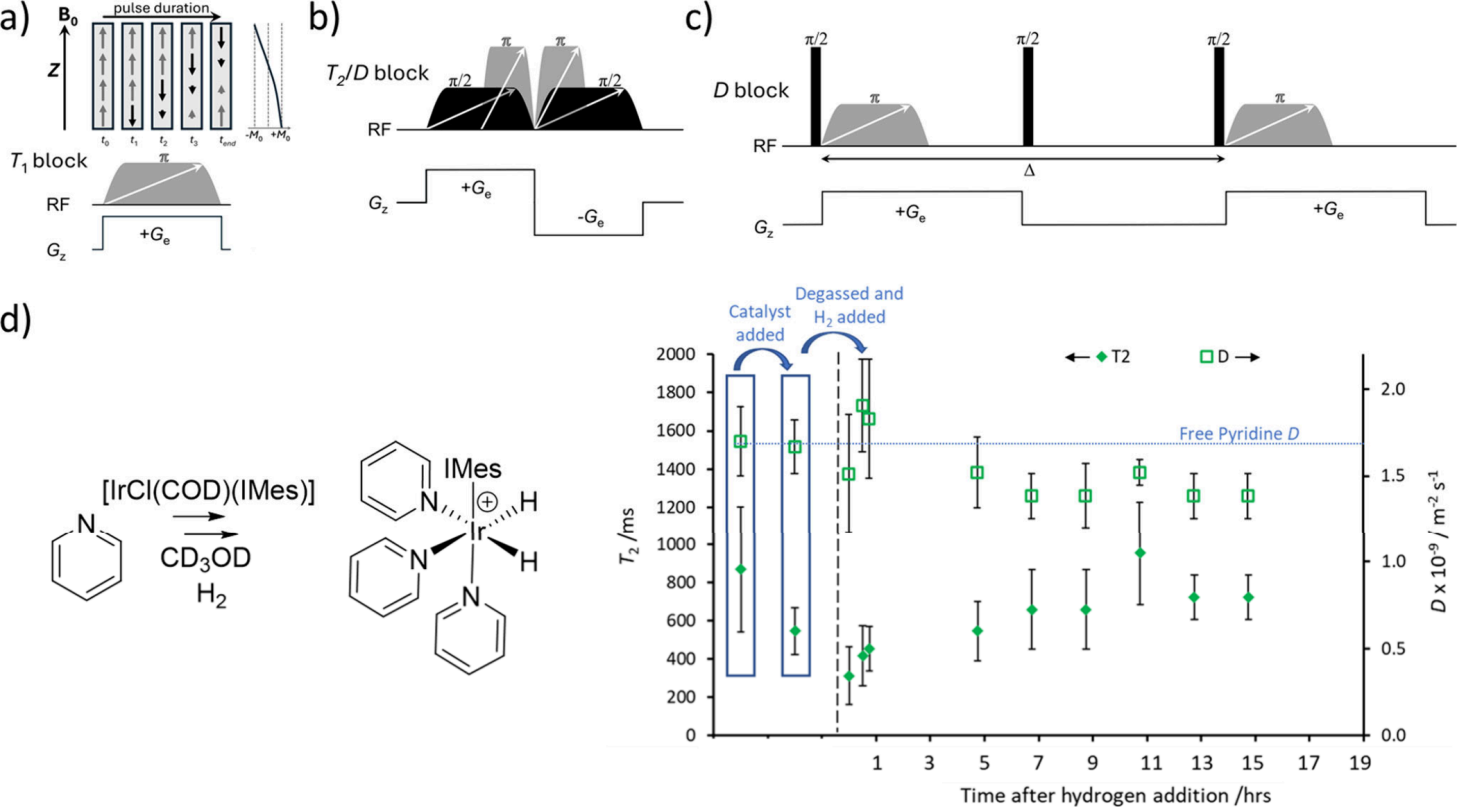
Examples of ultrafast Laplace NMR blocks used for spatial encoding of (a) *T*_1_, (b) *T*_2_, and (b,c) *D*. The upper part of a) illustrates the process of spatial encoding of *T*_1_ information into longitudinal magnetization during the frequency-swept π-pulse. The block in (b) can be used for both encoding of *T*_2_ and *D*, depending on the strength and duration of *G*_e_. the Δ parameter in (c) is equivalent to the diffusion time in conventional pulsed-field gradient NMR. An example of using spatially encoded ultrafast Laplace *D*-*T*_2_ correlation experiments to monitor the reaction of pyridine with an iridium catalyst is shown at 298 K and 9.4 T. In this reaction, binding of pyridine to the metal center is indicated by a drop in *D* and *T*_2_. Over longer time scales, the iridium-catalyzed incorporation of the deuterium label from the solvent methanol-*d*_4_ into pyridine is tracked by a lengthening of the pyridine *T*_2_. These proof of concept studies show the potential for these experiments to monitor chemical reactivity, although in this example the reactions can be easily monitored using ^1^H NMR spectroscopy. Panel (d) is reproduced and adapted with permission from ref (73). Copyright 2021, Royal Society of Chemistry.

NMR relaxation and diffusion experiments can offer additional chemical insight beyond spectroscopic information contained in resonance frequencies.^55,56^ Such experiments provide detailed information on molecular dynamics with *T*_1_ and *T*_2_ relaxation rates reflecting mainly rotational motion and diffusion coefficients, *D*, characterizing the stochastic translational motion. These methods are valuable for studying materials including various liquids, polymers, proteins, and more.^57−62^ Moreover, *T*_1_, *T*_2_, and *D* are sensitive to structural and environmental changes, making it possible to monitor the chemical transformations of the samples under study. The data from these experiments decay exponentially, and the distribution of relaxation times or diffusion coefficients can be determined through an inverse Laplace transform, categorizing these experiments as Laplace NMR. Ultrafast methods that exploit spatial encoding can also be used to collect ultrafast Laplace NMR relaxation and diffusion experiments.^63,64^ However, the pulse sequence building blocks in this case are different from Fourier-transform ultrafast NMR as chemical shift information is refocused while relaxation or diffusion-weighted magnetization profiles are created along the spatial dimension. Examples of such blocks for *T*_1_, *T*_2_, and *D* weighting are shown in Figure 5a–c.^64^ For instance, during the application of a *T*_1_ block (Figure 5a), longitudinal magnetization in different spatial locations, *M*_*z*_(z), will reflect the inversion–recovery curve. Therefore, reading the *M*_*z*_(z) profile using 1D imaging can provide *T*_1_ information in a single scan. Similarly, reading the longitudinal or transverse magnetization profiles after applying the blocks shown in Figure 5b and c can be used to obtain information about *T*_2_/*D* or *D*, respectively. Measured *T*_2_/*D* profiles will be dominated by either *T*_2_- or *D*-weighting, depending on the experimental parameters applied during this block.

In spatially encoded ultrafast Laplace NMR a CPMG loop readout is typically used to perform the 1D imaging detection of the resulting magnetization profiles by applying a read gradient during the sampling of the spin–echo signals.^64^ Therefore, the collected data are 2D by nature with indirect spatial and direct CPMG directions. It is worth noting that depending on the strengths of the read gradient, the CPMG echoes can also reflect *T*_2_ or *D* weighting. This provides the possibility to measure *X*-*T*_2_ and *X*-*D* correlation experiments in one scan, where X = *T*_1_, *T*_2_, or *D*. For example, these approaches were used to study porous media and hydrocarbon mixtures using *T*_1_-*T*_2_^65^ and/or *D*-*T*_2_^66^ correlations. Correlating the same parameters (e.g., *T*_2_-*T*_2_ and *D*-*D* experiments) can give information about molecular exchange. For instance, water exchange between vesicles and free solution was probed by *D*-*D* diffusion exchange spectroscopy (DEXSY)^67^ ultrafast experiments and similarly exchange of chemical components of ionic liquids between its aggregates was studied by a *T*_2_-*T*_2_^68^ relaxation exchange spectroscopy (REXSY) approach to provide exchange rate parameters. These methods are excellent at detecting physical changes, in particular, the movement of molecules, typically water, into pores in macroscale structures. As these changes are best revealed by changes in *T*_1_, *T*_2_, and *D*, they can be tracked using ultrafast Laplace NMR in a way that traditional 2D NMR cannot. Examples include examining water movement in silica^66^ and cement,^69,70^ the monitoring of cheese ripening,^71^ or monitoring the aggregation of ionic liquids.^68,72^ Regardless of evident potential, these methods have not yet broadly been applied to studying chemical reactions, although there is one example using changes in *T*_2_ and *D* to monitor binding of pyridine to an iridium catalyst and its metal-catalyzed deuteration (Figure 5d).^73^

## Combining Ultrafast NMR with Hyperpolarization

All NMR methods suffer from a low signal intensity. This stems from the radio frequency perturbation of closely spaced nuclear spin energy levels. As the population differences across these states, described by the Boltzmann laws, are small, the corresponding NMR signal intensities are also small. This sensitivity issue becomes even worse at lower magnetic fields and for heteronuclei with lower gyromagnetic ratios. Accordingly, ultrafast NMR, whether based on a Fourier transform or Laplace transform, also suffers from low sensitivity due to low thermal polarization, although there is an additional sensitivity penalty due to spatial encoding. Fortunately, in the last few decades a series of methods have arisen that have allowed NMR signal intensity to be boosted by many orders of magnitude.^74,75^ A family of methods have arisen which are termed hyperpolarization techniques and can generate NMR signals dramatically larger than those recorded using thermally polarized, Boltzmann controlled, NMR. Hyperpolarization is therefore used in applications including biomedical imaging, reaction monitoring, and low concentration analysis as the enhanced NMR signals facilitate detection of molecules with short lifetimes and low concentrations without signal averaging.^76−78^

NMR experiments used to detect hyperpolarized species can involve simple 1D detection or more complex 2D or higher dimensional spectroscopy. In these cases, signal averaging is often not required due to the boost in the NMR signal intensity provided by hyperpolarization. However, there are additional challenges with collection of 2D data sets associated with pulse-induced magnetization depletion. This is a consequence of the fact that hyperpolarized molecules are a nonequilibrium spin state that relax back to Boltzmann controlled populations. As such, the magnetization available for detection in the experiment is being effectively “used up” by successive radiofrequency pulses and decaying due to relaxation. This consideration does not apply to thermally polarized 2D NMR, in which constant steady-state magnetization can easily be achieved.

Generally, detection of hyperpolarized molecules can be either “single shot” which typically involves a single NMR experiment. Alternatively, “multishot” detection involves collection of multiple repeat NMR experiments separated by a rehyperpolarization step to replace the enhanced magnetization between each scan. This latter approach is analogous to conventional multiscan NMR in which steady state magnetization is used, albeit of much lower intensity than in “multishot” hyperpolarisation experiments.

Multishot 2D detection of hyperpolarized molecules using experiments such as conventional DOSY^79−81^ and HMQC^82^ have been employed and are particularly useful when combined with multiple/repeat hyperpolarization steps.^83^ In these cases, 2D detection of hyperpolarized species can be achieved without ultrafast experiments. However, implementation of these experiments can be restricted by the limitations of the hyperpolarization method, for which repeated steps may not be feasible. Therefore, the combination of ultrafast detection methods with “single-shot” (and to a lesser extent “multishot”) hyperpolarization methods is highly advantageous as they allow 2D data sets (such as COSY and TOCSY) to be collected in just a single scan. Marrying the two techniques together is very attractive, as it provides an application for ultrafast experiments to detect molecules that can be challenging to discern using conventional 2D NMR methods, and it also addresses a significant weakness of hyperpolarized 1D experiments by providing the extra resolution from 2D NMR, without the time penalty of recording indirect dimension points.

It is no surprise that in recent years several examples have arisen that use fast NMR methods for 2D detection of hyperpolarized species.^84−88^

This can include examples that use Hadamard encoding,^89,90^ or other sampling strategies^91^ to reduce experimental time, or spatial encoding to detect hyperpolarized species. In this Perspective, we focus on examples of spatially encoded single scan experiments for ultrafast NMR detection. For example, *D*-*T*_2_ correlation experiments are shown to facilitate single-shot detection of various hyperpolarized substances using PHIP,^66^ SABRE,^73^ SEOP,^92^ and dDNP^32,66,93−95^ hyperpolarization techniques. Some examples have gone further by using these types of experiments to detect physically or chemically changing hyperpolarized molecules. For example, the target benzylamine was hyperpolarized using dDNP to boost its ^13^C NMR signals by a factor of 4,500 to 5,800-fold.^96^ The binding of this target to the enzyme trypsin was then studied using ultrafast NMR by recording a series of *T*_2_ relaxation experiments that correlate spatially encoded chemical shift with *T*_2_ relaxation in experiments that take milliseconds per scan. Accordingly, information about the binding of the ligand to the protein could be determined from changes to the *T*_2_ relaxation time. Notably, this binding could not be probed using traditional ^1^H or ^13^C NMR, even with hyperpolarization, as no significant change in the chemical shift is observed upon binding. Therefore, these kinds of experiments are highly useful for examples where sample change is only detectable *via* changes in *T*_1_, *T*_2_, or *D*, which can take significant time (greater than tens of minutes) to record using traditional 1D experiments.

**Figure 6:**
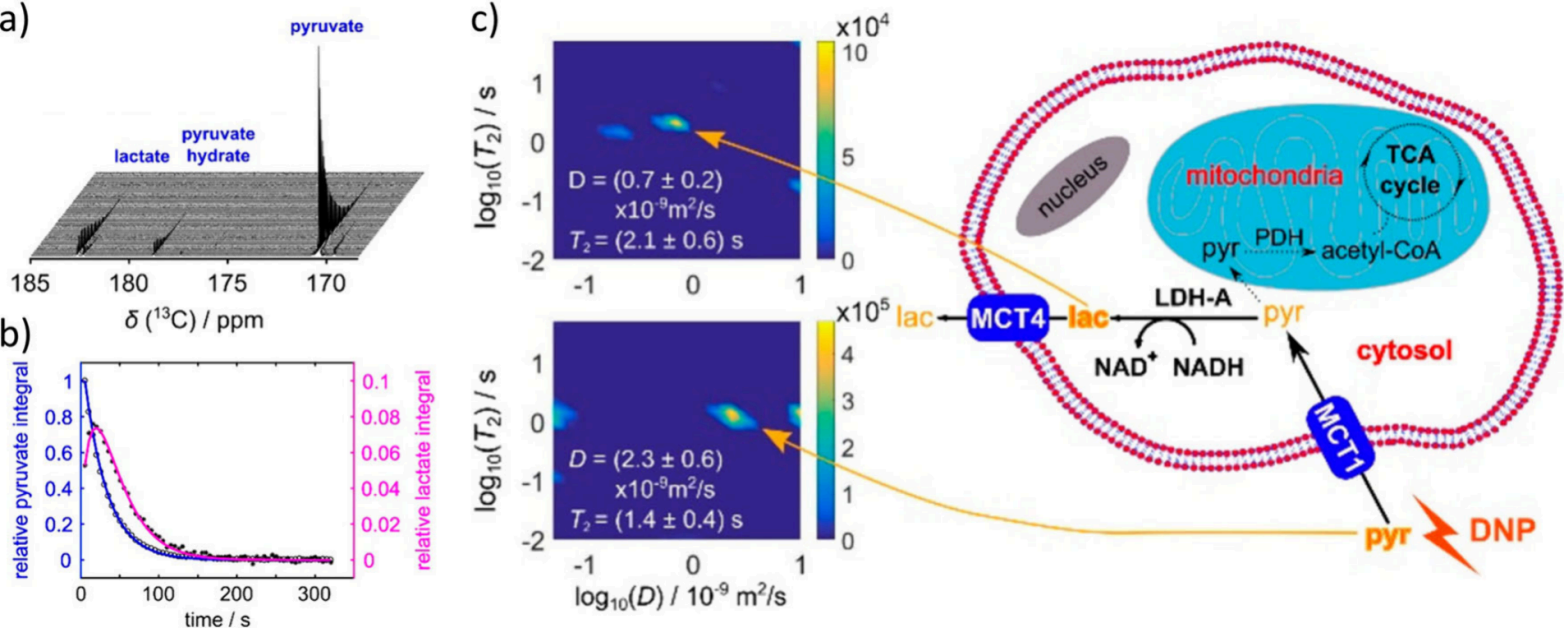
Example of ultrafast hyperpolarized Laplace NMR to detect the reaction of pyruvate to lactate in 4T1 mice cancer cells. ^13^C-pyruvate hyperpolarized using dDNP was injected into a cell culture and the resulting enzymatic pyruvate to lactate conversion was monitored using (a) a series of small flip-angle ^13^C NMR spectra. (b) Time course derived from hyperpolarized 1D ^13^C NMR spectra. (c) Species selective ultrafast ^13^C *D*-*T*_2_ experiments made it possible to conclude that the hyperpolarized pyruvate and its metabolic product lactate belonged predominantly to different intra- and extracellular compartments. Reproduced and adapted with permission from ref (93). Copyright 2018, American Chemical Society.

Similarly, ultrafast Laplace NMR has also been used to monitor changes in a hyperpolarized target. A rare example used dDNP to hyperpolarize ^13^C-pyruvate and inject it into cell cultures of mouse 4T1 cancer cells.^93^ The hyperpolarized ^13^C-pyruvate could be detected using ^13^C *D*-*T*_2_ experiments, and its metabolic product lactate could also be detected in these experiments (Figure 6). This pyruvate to lactate conversion, which is upregulated in areas of cancer, is often used in hyperpolarized imaging to localize areas of cancer.^97^ However, this is typically identified based on changes in the ^13^C chemical shift, which is often sufficient to allow this reaction to be monitored. However, a weakness of current methods is that by overlaying a ^13^C–CSI over a ^1^H anatomical image, the diagnostic ^13^C information is one-dimensional in nature. In contrast, the advantage of probing this reactivity using ultrafast *D*-*T*_2_ is that the *D* and *T*_2_ values allow the pyruvate and lactate to be correlated to intracellular or extracellular environments.

## Conclusions

In this Perspective, we have outlined the principles behind ultrafast NMR and given examples of how it has been applied to monitor specific chemical reactions. Currently, ultrafast NMR is typically used by research institutions that have a high level of magnetic resonance expertise. Ultrafast experiments are not standard pulse sequences, they are typically custom-made and home-written which is often time-consuming and a significant barrier to their more widespread use. Going forward, there will be many developments in specific ultrafast sequences to improve speed, resolution, and implementation. We expect that in the years ahead these sequences will become more accessible to nonexperts. We hope our perspective inspires a wider range of synthetic chemists to apply ultrafast NMR experiments to their own systems, particularly in those cases where traditional NMR suffers from peak overlap or long acquisition times. Ultrafast NMR is such a powerful tool for probing fast chemical transformations, and we expect this method will become a more widely used and mainstream approach in the coming years.

## Abbreviations

COSY: correlation spectroscopy
CPMG: Carr Purcell Meiboom Gill
dDNP: dissolution dynamic nuclear polarization
DEXSY: diffusion exchange spectroscopy
DQ: double quantum
EPSI: echo planar spectroscopic imaging
FOV: field of view
HMBC: heteronuclear multiple bond correlation
HSQC: heteronuclear single quantum coherence
MR: magnetic resonance
MRI: magnetic resonance imaging
NMR: nuclear magnetic resonance
NOE: nuclear Overhauser effect
PHIP: *para* hydrogen-induced polarization
REXSY: relaxation exchange spectroscopy
SABRE: signal amplification by reversible exchange
SEOP: spin exchange optical pumping
SOFAST: band-Selective Optimized Flip-Angle Short-Transient
SQ: single quantum
TOCSY: total correlation spectroscopy
UF: ultrafast
1D: one-dimensional
2D: two-dimensional
